# Unraveling the intricate molecular mechanisms of sex hormones’ roles in breast cancer pathogenesis: A review

**DOI:** 10.1097/MD.0000000000049376

**Published:** 2026-06-26

**Authors:** Matthew Chibunna Igwe, Ogbonna Alphonsus Ogbuabor, Simeon Ikechukwu Egba

**Affiliations:** a Department of Public Health, School of Allied Health Sciences, Kampala International University, Kansanga, Uganda; b Department of Medical Laboratory Science, Faculty of Basic Medical Sciences, College Medicine, Enugu State University of Science and Technology, Enugu, Nigeria; c Department of Biochemistry, Research and Extension. Kampala International University, Kansanga, Uganda.

**Keywords:** advanced, breast cancer development, sex steroids, structure–function relationships peculiarities

## Abstract

This study examines the role and function of the specific signaling molecules that are associated with the binding of sex hormone receptors, including estrogen receptor (ER) and progesterone receptor (PR), in relation to cell proliferation, differentiation, and survival. The present work focuses on the mutual relationship between signaling pathways of sex hormone receptors and MAPK and PI3K/AKT pathways, as well as the involvement of these molecules in the development of breast cancer. The review systemizes the present knowledge regarding genetic changes related to breast cancer, including the effects of changes on sex hormone signaling, biochemical pathways influencing sex hormone availability, and epigenetic modifications. The focus is made on genetic changes the researchers observed in breast cancer, like ERBB2/HER2 amplification and TP53 mutations, which cooperate with sex hormone signaling to advance tumor growth. The changes in the metabolism of sex hormones are as follows: High aromatase leading to high estrogen and more. Furthermore, the impact of sex steroids on the tumor microenvironment, including angiogenesis, immune regulation, and matrix, is consequently discussed. The effects of sex hormones on DNA methylation, histone modifications, and noncoding RNA with regard to breast cancer development are reviewed. The study also demonstrates how genetic changes, metabolism, and epigenetics work in concert with sex hormones to modulate breast cancer development. SERMs, aromatase inhibitors, and the endocrine approach are described, as well as novel directions to develop inhibitors of androgen and other hormone signaling pathways.

## 1. Introduction

Being the second most common cancer and the most frequent cause of cancer-related morbidity and mortality in women globally, breast cancer is still a heterogeneous, poly-factorial process dependent on genetic, hormonal, and environmental factors.^[1]^ Among the above- described contributors, sex hormones, mainly estrogen and progesterone are the first ones that are critical to breast cancer development since they bind to their receptors – ER and PR – and affect processes like proliferation, differentiation, as well as cell survival.^[2]^ As these hormones play important roles in tumor initiation and progression, their specific molecular interactions remain an important and active line of research because it may impact hormone-specific therapies and idiosyncratic treatment options.

The research of recent years has appointed concrete chains of several types of sex hormone receptors and their activation: MAPK and PI3K/AKT dependent on them, which control the growth of cells, their death, and metastasizing.^[3,4]^ In addition, other genetic changes that commonly occur in breast cancer with the signaling pathways controlling hormone response contribute towards the aggressiveness of the disease and the tendency to exhibit multidirectional responses to therapies. Despite remarkable advances in the molecular discipline of these interactions, there are still lacunas in knowledge about how various metabolites and other epigenetic changes affect hormonal activity. For instance, it was reported that dysregulated activity of aromatase which leads to increased estrogen synthesis is pro-tumorigenic.^[5,6]^ However, hormonal signaling-independent epigenetic changes reflected in DNA methylation, histone

modifications, noncoding RNA expression alterations add extra layers to breast cancer complexity.^[7]^

This review hypothesizes that male–female sex hormones intermittently modulate genetic, metabolic, and epigenetic influences, and that these are prime determinants of breast cancer heterogeneity and progression. To address this hypothesis, we aim to: identify the signaling pathways of sex hormone receptors in breast cancer and their physiological consequences; assess the pivotal genetic/epigenetic changes in regard to hormone-driven tumor progression; comprehend how metabolic modifications influence hormone availability and efficacy; and discuss the current and potential treatments targeting sex hormone receptors. In bridging these gaps in knowledge, our objective is to gain a common understanding of the molecular basis of sex hormone signaling and the growth of breast cancer, and thus better direct treatment regimens and outcomes for patients.

## 2. Epidemiological context

Breast cancer remains a major global health challenge, with an intricate and multifactorial pathogenesis shaped by genetic, hormonal, and environmental influences. The exploration of sex hormones in breast cancer epidemiology reveals a dynamic interface that contributes to disease onset, progression, and outcomes across diverse populations. Epidemiological data highlight the global burden of breast cancer, with regional variations in incidence rates often reflecting differences in hormonal exposures and lifestyle factors common in developed versus developing regions.^[8]^ Higher prevalence rates in developed countries underscore the influence of lifestyle factors such as obesity, reproductive patterns, and exogenous hormone use, all of which modulate hormonal activity.^[9]^

Breast cancer is categorized into subtypes based on hormone receptor status – ER-positive, PR-positive, HER2-positive, and triple-negative – with each exhibiting distinct clinical behaviors and epidemiological patterns.^[10]^ This differentiation has profound implications for understanding disease risk, prognosis, and therapeutic response. For instance, postmenopausal obesity has been linked to an increased risk of hormone receptor-positive breast cancer due to the role of adipose tissue in estrogen synthesis, highlighting the complexity of hormonal influence on disease etiology.^[11]^ Furthermore, exogenous hormone use, including oral contraceptives and HRT, demonstrates subtype-specific associations, further emphasizing the importance of tailored epidemiological and clinical assessments.^[12]^

Reproductive factors such as early menarche, late menopause, parity, and breastfeeding duration impact breast cancer risk by modulating hormonal exposure over a lifetime.^[13]^ These factors predominantly influence hormone receptor-positive subtypes, contributing to an elevated risk among women with extended periods of hormonal stimulation. Genetic susceptibility also plays a crucial role; specific genetic variants affecting hormonal pathways have been identified, deepening our understanding of individualized risk profiles.^[14]^

Geographic and socioeconomic disparities significantly shape hormonal risk factor exposure, with lifestyle, environmental, and healthcare access differences contributing to diverse epidemiological landscapes.^[15]^ Endocrine-disrupting chemicals further complicate the hormonal milieu, potentially mimicking or interfering with normal hormonal signaling and contributing to breast cancer risk.^[16]^ The impact of hormonal fluctuations during pregnancy and their long-term implications for breast cancer risk is another area of active investigation, highlighting the influence of reproductive history on disease development.^[17]^

Physical activity is shown to modulate hormonal levels, with epidemiological studies linking exercise-induced changes to reduced breast cancer risk, offering a holistic preventive strategy.^[18]^ Disruptions in circadian rhythms, which influence melatonin production, further underscore the complex relationship between hormonal regulation and breast cancer risk.^[19]^ Finally, the interplay between sex hormones and the immune microenvironment plays a pivotal role in breast cancer pathogenesis, emphasizing the need for comprehensive investigations into how hormonal fluctuations impact immune responses.^[20]^

The purpose of this review is to present a clear picture of the role of sex hormones in breast cancer and its epidemiological perspective for understanding genomic variations that lead to disease heterogeneity and to present research gaps for more focused therapeutic approaches.

## 3. No statutory risk factors for breast cancer

It is noteworthy that the factors predisposing to the development of breast cancer are numerous and occupy genetic, lifestyle, reproductive, and environmental levels. The gender most at risk is female since the occurrence of the condition makes the risk relative to males 100 times higher.^[21]^ Although a majority of breast cancer cases are not thought to be related to the inheritance of cancer gene mutations, genetic factors do underlie a subset of the disease and are known to significantly affect disease risk and age of onset. These mutations remain the most widely understood gene defects predisposing women to hereditary breast cancer; these women have up to a 70% probability of developing tumors before they reach 80 years of age.^[22]^ Such a mutation increases overall risk and more often results in a younger age at diagnosis in comparison to noncarriers.^[23]^

In addition to BRCA mutations, several less common genetic variants have been identified, though their impact on breast cancer risk is generally more modest.^[24]^ Examples include mutations in PALB2 (BRCA2 partner and localizer), CHEK2 (checkpoint kinase2), and ATM (ataxia telangiectasia mutated), which contribute to moderate increases in risk compared to BRCA1/2 mutations.^[25]^ Importantly, familial history of breast cancer amplifies risk beyond genetic predispositions, suggesting potential interactions between inherited genetic factors and shared environmental or lifestyle exposures.^[26]^

Another source of risk is breast tissue density – breast tissue in women with dense breasts is difficult to detect during the early stages, and this group might also be at a higher risk because breasts are derived more from glandular tissue than fatty tissues.^[27]^ Breast cancer etiology is further made complex by hormonal activities since some environmental factors, like endocrine-disrupting chemicals, are reported to modulate or mimic hormonal signaling pathways.^[28]^ Hormonally susceptible, these exposures participate in a milieu which not only increases disease risks in genetically vulnerable folks but also augments the consequences of a victim’s preexisting susceptibilities.

### 3.1. Breast cancer subtypes

Both hormone receptor status and gene expression profiles define breast cancer into unique molecular subtypes such as Luminal A, Luminal B, HER2-enriched, and TNBC. These subtypes have implications for prognosis, disease progression, and treatment.^[29]^ Luminal A tumors are ER/PR positive and have low proliferative activity, as noted by a low Ki-67 Index. This subtype is generally thought to be less aggressive, and patients usually have better survival rates and are sensitive to endocrine therapy.^[30]^

Luminal B tumors share the characteristic of ER/PR positivity but differ from Luminal A tumors in having higher Ki-67, which points to a higher proliferative activity of such tumors and, therefore, a higher clinical invasiveness.^[31]^ Higher proliferation rates also predict poorer outcomes, an increased risk of early relapse, and the need for more intense treatment, which may be endocrine and/or chemical.^[32]^

HER2-enriched breast cancer is defined by the overexpression of HER2, which drives aggressive tumor behavior through enhanced growth and survival signaling pathways. HER2− positive tumors often exhibit rapid proliferation and are associated with poorer outcomes when untreated. However, targeted therapies, such as trastuzumab and pertuzumab, have demonstrated significant clinical efficacy in improving outcomes for patients with this subtype.^[33]^

Basal-like or TNBC is characterized by the lack of ER, PR, and HER2 and the disease carries a poor prognosis due to the dearth of therapies targeted at this molecular subtype.^[34]^ TNBC shows basal epithelial cell features and, therefore, is more invasive, recurs more often, and has worse outcomes. Other treatment methods, such as chemotherapy, immunotherapy, and later molecular-targeted therapy, are being sought to enhance favorable prognosis for this tough subtype.^[35]^

### 3.2. ER signaling and the neoplastic phenotype in breast cancer

The hormonal pathway that is most critical in breast cancer is the estrogen receptor/transcription factor signaling pathway because about 50% to 70% of breast cancers are ER-positive. When binding to estrogen, ER orchestrates complex signaling cascades that regulate gene expression linked to cell proliferation and survival, significantly influencing breast cancer progression.^[36]^ ERs come in 2 major families, ERα and ERβ, both of which, when bound to estrogen, alter conformation, dimerize, and translocate to the nucleus. In the nucleus, they perform the duties of a transcription factor involved in the regulation of gene activity.^[37]^

ERs are involved in the activation of the transcription of pertinent target genes important in cell cycle control, cell death, and really repair. Some examples are cyclin D1, a cell cycle protein that propels the cycle; Bcl-2, an antiapoptotic protein; and p53 a protein responsible for tumor suppression that also functions in DNA damage.^[38]^ ER-positive breast cancers which have viable, functional ERs can often be treated with SERMs and aromatase inhibitors that react with estrogen.^[39]^ But long-term activation of ER signaling may make it possible for cells to divide uncontrollably, thus leading to the formation of tumors and progression of ER-positive breast cancers.^[40]^ There is still the emerging problem of endocrine resistance in the treatment of ER-positive breast cancer patients. Mechanisms of resistance include mutations in the ER gene (ESR1), changes in co-regulator expression, and interaction with growth factor signaling pathways such as HER2 that activate other pathways incompatible with the requirement for estrogen.^[41]^ Efforts are being made to unlock endocrine resistance, and these include modulation of downstream ER signaling and development of SERD. SERDs act by destabilizing and degrading ER, thus inhibiting its transcriptional activity and presenting a novel strategy against resistance.^[42]^

### 3.3. Progesterone-mediated effects in breast cancer development

The present review suggests that progesterone has a critical involvement in the biology of breast tumors and impacts cellular events in ways beyond the traditional conception of its roles in conception and lactation. Both of the known isoforms of PR are PR-A and PR-B that regulate numerous and multifaceted effects of progesterone in breast cancer cells. The cross-talk between progesterone and estrogen signaling pathways is found to have a complex hormonal regulation of breast cancer.^[43]^ Following ligand binding, PR appears to undergo conformational changes that enable dimerization and nuclear translocation where this receptor acts as a trans-activator regulating gene expression.^[44]^

That progesterone is essential for the proper growth of the mammary gland, mainly in the process of alveolar transformation and lobuloalveolar proliferation during pregnancy. During PR activation, induction of cell proliferation and differentiation enhances the mammary epithelial cell and prepares the gland for lactation.^[45]^ Nevertheless, chronic administration of progesterone can also be involved in the initiation and progression of breast cancer, especially within the frame of hormone receptive positive subtypes by supporting the mechanisms of cell division and survival.^[46]^

In hormone receptor-positive breast cancers, progesterone works cooperatively with estrogen in regulating the reaction to endocrine medications and the actions of the cancer itself. In detail, PR can partially agonize the function of ER; however, the cancers that have both ER and PR are likely to have different prognoses.^[47]^ The 2 isoforms, PR A and PR B, can exert opposite or qualitative effects on a target gene, and thus contribute to the modulation of cellular responses by progesterone in breast cancer.^[48]^

## 4. Genetic change in breast cancer

### 4.1. Erythroblastic oncogene B/human epidermal growth factor receptor 2 (ERBB2/HER2) amplification

In breast cancer, ERBB2/HER2, which encodes a receptor tyrosine kinase, is one of the most often amplified genes. This amplification leads to overexpression of the HER2 protein on the surface of the cancer cell, increasing the signaling that initiates the cancer process. More importantly, it has been found that ERBB2/HER2 amplification cooperates with sex hormone signaling, especially estrogen receptor signaling, to enhance tumor initiation and progression.^[49,50]^

Still, it has been identified that several molecular mechanisms regulate the connection between ERBB2/HER2 amplification and sex hormone signaling. One of the mechanisms is through crosstalk between ERBB2 and ER to facilitate signaling by other molecules involved in cell division, cell survival, and cell motility. This crosstalk is often through c-SRC, a non-receptor tyrosine kinase central to signal transduction. ERBB2/HER2 enhances c-SRC, and in turn, PRAS and PRAS- bound ERα is phosphorylate at series 105/110, 118, 167/169, and 421/423 to amplify the transcriptional activity. This phosphorylation process positively regulates the gene expression of targets that belong to ER, and these targets are involved in cell growth and survival.^[51,52]^

In addition, ERBB2/HER2 overexpression can affect ER function by triggering downstream signaling, including through the PI3K/AKT pathways. This activation leads to phosphorylation and thus activation of transcription factors such as FOXO3A and GATA 3 which are all essential regulators of ER signaling that promotes the proliferation of breast cancer cells.^[53]^ There is also an upregulation of ERBB2/HER2 signaling, which affects the level and function of the coregulatory proteins for ER. In particular, HER2 increases the level of coactivators such as AIB1 (amplified in breast cancer 1) and PELP1 (proline, glutamic acid, and leucine-rich protein 1) and stimulates ER signaling, thus promoting hormone independence in breast cancer cells.^[54,55]^

### 4.2. Tumor protein p53 (TP53) in breast cancer

Aberrations in TP53 are among the most frequent genetic changes identified in breast cancer; these changes affect the regulation and function of the sex hormone receptors. As one of the tumor suppressor genes, TP53 gives instructions for p53 protein, which is important for the stability of a genome and avoiding the development of cancer. On the other hand, loss or dysfunction of p53 due to mutations in the TP53 gene usually contributes positively to tumorigenesis and cancer progression.^[45,56]^

One of the ways by which TP53 mutations are believed to influence sex hormone receptors is by interacting with ERα. Under normal conditions, it is possible for p53 to bind physically to the promoter of the ERα gene so as to facilitate its transcription. Reduced functional p53 through TP53 mutations has been found to have an inhibitory effect on estrogen receptor-α-mediated signaling that is important in print hormone receptor-positive breast cancer.^[57]^ This dysregulation can result in changes in cellular signaling towards estrogen and can affect the behavior of breast cancer and response to treatment.^[58]^

Moreover, TP53 mutations affect the activity of coactivators and corepressors of sex hormone receptors in particular. For example, it has been reported that p53 interacts with some transcriptional corepressors such as NCoR and silencing mediator for retinoid and SMRT. Mutant p53 can cause altered ratios between coactivator proteins and corepressor proteins, enabling aberrant sex hormone receptor signaling in breast cancer.^[59]^

In addition, the loss of TP53 affects DNA repair, which is crucial for maintaining genome stability. They also note that the p53 protein has a critical position in the DNA damage response, controlling genes including p21 and GADD45 that are engaged in DNA repair. Because p53 is involved in the process of DNA repair, its dysfunction caused by TP53 mutations leads to the formation of new genetic changes in breast cancer, including alterations of the hormonal receptor signaling pathways.^[60]^

## 5. Metabolic conversion of sex hormones in breast cancer development

The metabolic transformations of sex hormones are central to regulating their bioavailability and maintaining hormonal equilibrium. These processes involve various enzymatic reactions across different tissues, such as the liver, kidneys, and adipose tissue. A deep understanding of the molecular mechanisms underlying these transformations is critical for elucidating the regulation of sex hormone levels and their implications in breast cancer pathogenesis.^[44]^

One prominent metabolic transformation involves the conversion of sex hormones between their active and inactive forms. For instance, estrogens like estradiol can be metabolized into less active forms, such as estrone and estriol, through hydroxylation. This reaction is primarily mediated by cytochrome P450 enzymes, including CYP1A1, CYP1B1, and CYP3A4. Such metabolic conversions reduce estrogenic activity, influencing hormone availability and action within tissues.^[51,61]^

Other catabolism pathways include conjugation, which adds products such as glucuronic acid, sulfate, or glutathione to sex hormones. It selectively occurs in the liver and is highly induced by various enzymes, including UDP-glucuronosyltransferases (UGTs) and sulfotransferases (SULTs). This stereochemistry increases the aqueous solubility of sex hormones and their elimination through urine or bile. Thus, by reducing the number of hormones to which the organism is exposed, these reactions act as a main feedback control in hormone balance.^[62]^

Furthermore, oxidative metabolism, where cytochrome P450 enzymes similar to CYP17A1, CYP19A1, and CYP3A 4 acts to synthesize and catabolize androgens such as testosterone and dihydrotestosterone, and estrogens such as estradiol. This metabolic process can produce active metabolites or, in some cases, inactive hormones. Apparently, there is a rather delicate equilibrium between these metabolic processes to ensure the right hormonal level and their functioning.^[63]^ Dysregulation of these metabolic pathways may cause hormonal abnormalities and contribute to the generation of hormone-associated diseases, such as breast cancer, due to the change in hormone signal dynamics.^[8]^

## 6. Sex hormone receptor expression and tumor environment

The effects of sex hormones on the TME are far-reaching, and the underlying molecular pathways that are altered are diverse. Their function may impact several aspects of tumor development. Such mechanisms include the repression of pro-inflammatory cytokines, angiogenesis, immune system regulation, and remodeling of the tumor microenvironment.^[37]^ Knowledge of the TME’s influences by sex hormones is crucial for designing therapies with a focus on the interference of these processes in breast cancer.^[12]^

Sex hormones, particularly estrogen and progesterone, exert their effects on the TME by binding to their respective receptors, ER and PR, expressed on both cancer cells and other cellular components within the TME. The activation of these receptors triggers intracellular signaling cascades that influence gene expression, cell proliferation, and cellular behavior.^[64]^ ER signaling has also been reported to enhance the secretion of VEGF (vascular endothelial growth factor), which brings about the development of blood vessels supplying the tumor.^[65]^ Moreover, estrogen participates in the regulation of inflammation in the TME through the alteration of levels of pro-inflammatory cytokines, including IL-6 (interleukin-6) and TNF-α (tumor necrosis factor-alpha), that promote tumor growth.^[66]^

Furthermore, these hormones can affect immune response and thus modulate and influence the TME. The immune cells affected by estrogen are, for instance, T cells, B cells, natural killer cells, and macrophages. It can alter both the phenotype and the behavior of immune cells, key aspects of the immune response, including migration and cytokine secretion, and could therefore act on immune responses in ways that either promote or inhibit the recognition of tumors and consequent destruction by adaptation.^[67]^ Sex hormones can also affect immune checkpoint proteins like PD-1 (apoptosis related protein 1) and CTLA-4 (cytotoxic T lymphocyte associated protein 4) related to immune cell activation that can otherwise suppress tumor immunity.^[61,68]^

Moreover, sex hormones are directly involved in the modulation of tissue factors and the activity of MMPs, which are enzymes involved in the degradation of ECM (extracellular matrix) in the TME. It has been reported that estrogen is capable of increasing the synthesis of certain MMPs for tissue remodeling, invasion, and metastasis of tumors.^[50]^ This process has decisively affirmed the fact that estrogen acts as an aggressive factor with regard to tumor progression by increasing the invasive potentials of the carcinogenic cells.

It is important to recognize that the effects of sex hormones on the TME can vary depending on several factors, including cancer subtype, hormone receptor status, and other molecular characteristics. As such, further research is needed to understand the context-specific roles of sex hormones in breast cancer progression.^[69]^ Such knowledge could result in improved pharmacotherapy in the hormonal manipulation of the TME.

## 7. Epigenetic modifications in hormone-responsive genes

Epigenetic alterations exhibit a vivid function in developing the response of breast cancer cells to hormonal signals by DNA methylation, histone changes, and chromatin remodeling. Methylation, one of the major epigenetic modifications, occurs at CpG dinucleotides mostly in gene-promoting regions, and involves the addition of 1 or more methyl groups to the cytosine residues. This modification can cause the repression of hormone-dependent genes in the arrest of transcription factor alkylation.^[29]^

Acetylation, methylation, and phosphorylation of histones and other chromosomal proteins play an important role in the regulation of gene chromatin structure and hormonal response. For instance, while histone acetylation usually reveals genes in euchromatin to the general transcription machinery, histone hypoacetylation condenses them in heterochromatin. Conversely, histone methylation can be either transcription activating or transcription silencing, depending on the nature of methyl group addition and the location of the modification. New studies have shown that DNA methylation and histone modifications are not always independent; thus, the 2 complicate the progression of breast cancer and the development of resistance to therapies.^[59]^

The involvement of noncoding RNAs, including microRNAs and long noncoding RNAs, is considerable in the regulation of hormone-responsive genes. MicroRNAs can bind mRNAs of hormone-responsive genes and either promote their degradation or inhibit their translation and affect the ability of cells to respond to hormones.^[70]^

Epigenetic modifications, especially those associated with hormone-responsive breast cancer, bear important implications on gene expression as well as on cancer development pathways. Erratic DNA methylation and histone modification could lead to the epigenetic inactivation of tumor suppressor genes and the activation of oncogenes controlling the growth, survival, and chemosensitivity of cancer cells.^[71,72]^ Knowing such intricate molecular processes is crucial for the creation of new therapeutic approaches to overcome the issues regarding the appearance of resistance to traditional endocrine therapies in breast cancer.

miR-21 (oncogenic miRNA): Suppresses apoptosis and stimulates cancer cell proliferation through PI3K/AKT signaling pathway Concerning lncRNAs such as HOTAIR, they interact with chromatin-modifying enzymes thereby affecting transcription products and the aggressive growth of cancer cells circRNAs act as RNA sponges thus modulating pathways like MAP kinase noncoding RNAs are involved in cancer development: diagnostic and therapeutic markers.^[13]^

## 8. Molecular mechanisms and signaling pathways

Sex steroid hormones – estrogen and progesterone – modulate breast cancer pathogenesis by interacting with their respective nuclear receptors: estrogen receptor alpha (ERα) and progesterone receptor (PR). These interactions activate a repertoire of transcriptional and non-transcriptional signaling mechanisms. Understanding these molecular dynamics is essential for identifying oncogenic targets and overcoming endocrine resistance. Estrogen signaling, predominantly via ERα, governs a wide array of cellular functions in breast tissue, including proliferation, apoptosis suppression, angiogenesis, and metabolic reprogramming. Two major modes of ERα signaling exist: genomic (nuclear) and non-genomic (extranuclear).

### 8.1. Genomic pathway

In the genomic pathway, 17β-estradiol (E2), the most potent endogenous estrogen, passively diffuses through the lipid bilayer and binds to cytoplasmic or nuclear ERα. Upon ligand binding:

ERα undergoes conformational changes, dimerizes, and is translocated to the nucleus; the receptor dimer binds to estrogen response elements (EREs) within promoter or enhancer regions of target genes.

This ER–ERE complex recruits transcriptional coactivators, including: SRC-3 (steroid receptor coactivator-3/NCOA3): Amplifies transcriptional activity via chromatin remodeling and interaction with p160 coactivator family; and CBP/p300: Histone acetyltransferases (HATs) that acetylate histones H3 and H4, loosening chromatin for transcription machinery access.^[73,74]^

Key target genes transcriptionally activated include:

1. CCND1 (Cyclin D1) – promoting G1/S cell cycle progression;
2. MYC – enhancing ribosome biogenesis and metabolic flux;
3. BCL2–inhibiting intrinsic apoptotic signaling.^[75]^

ERα activity is further modulated by posttranslational modifications, particularly phosphorylation at Ser118 and Ser167 (by MAPK and AKT, respectively). These modifications enhance ERα’s transcriptional potency even in low-estrogen environments; promote ligand-independent activation, contributing to endocrine resistance.^[76,77]^

### 8.2. Non-genomic pathway

Unlike genomic mechanisms that take hours to manifest, non-genomic estrogen signaling is triggered within seconds to minutes and operates independently of direct DNA binding. This rapid signaling relies on estrogen’s ability to engage membrane-associated receptors, activating downstream pathways that profoundly influence breast cancer cell behavior.

A subset of classical estrogen receptor alpha (ERα) localizes to the plasma membrane through S-palmitoylation at cysteine 451, which enhances its hydrophobicity and promotes interaction with caveolin-1 within lipid rafts.^[78]^ This membrane-associated ERα (mERα) forms complexes with Src family kinases, PI3K, and EGFR, initiating downstream signaling cascades. Such membrane localization of ERα is essential for rapid kinase activation, calcium mobilization, and nitric oxide release.^[79]^

GPER1 (GPR30) is a 7-transmembrane G-protein-coupled receptor that binds estrogen with high affinity and is prominently expressed in breast cancer cells – both ER-positive and TNBC subtypes. Activation of GPER1 triggers rapid increases in intracellular calcium and phosphatidylinositol (3,4,5)-trisphosphate, and mobilizes G protein–dependent signaling pathways. Cytoplasmic versus nuclear localization of GPER1 correlates with distinct prognoses and endocrine therapy outcomes.^[80]^

### 8.3. Downstream signaling cascades

Upon ligand binding, both mERα and GPER1 activate 3 main pathways: Activation leads to phosphorylation of AKT, promoting cell survival, antiapoptotic signaling, and nutrient-driven anabolic growth via mechanistic target of rapamycin (mTOR) activation – key to metabolic reprogramming and protein synthesis. This cascade is rapidly initiated and does not require transcriptional events.^[81]^ ERK1/2 activation stimulates proliferation, migration, and epithelial-to-mesenchymal transition (EMT). ERK can phosphorylate ERα Ser118, creating a feedback loop that enhances genomic ERα activity.^[82]^ Elevated cAMP from GPER1 signaling activates PKA, which phosphorylates CREB and other transcription factors, modulating gene expression indirectly and rapidly.^[83]^

Non-genomic pathways rapidly intersect with receptor tyrosine kinases (RTKs) such as HER2, EGFR, and IGF-1R, forming a ligand-independent network that sustains ERα activity and supports endocrine therapy resistance. Moreover, tamoxifen can upregulate GPER1, leading to enhanced activation of EGFR/MAPK pathways and reduced therapeutic efficacy.

GPER1 is highly expressed in TNBC and is implicated in EMT, invasion, and metastasis, driven by MAPK/EGFR activation. Genetic ablation of GPER1 in TNBC cell lines has shown increased apoptosis via JNK/p53/Noxa upregulation, highlighting its role as a therapeutic vulnerability.^[84,85]^

#### 8.3.1. Therapeutic implications

Advanced agents targeting membrane ERα palmitoylation, GPER1 antagonists (e.g., G15/G36), and kinase inhibitors are in preclinical development. Novel modalities like GPER1-targeted degraders (e.g., LYTACs or PROTACs) hold promise for blocking non-genomic estrogen signaling and circumventing endocrine resistance.^[86]^

#### 8.3.2. Dual functional roles of PR isoforms

PR-B, upon ligand binding (progesterone or synthetic progestins), activates transcription of proliferation-associated genes, including *CCND1*, *EGFR*, and Wnt family ligands; promotes stemness, epithelial proliferation, and angiogenesis, contributing to luminal tumor aggressiveness.^[43]^

Conversely, PR-A suppresses estrogen signaling by sequestering ERα coactivators or recruiting corepressors like NCoR1 and SMRT, which impairs estrogen-dependent gene transcription, thus potentially modifying endocrine therapy response.^[87,88]^ This antagonistic interaction between PR-A and PR-B creates a context-dependent hormonal response, influencing tumor phenotype, grade, and therapy responsiveness.

#### 8.3.3. PR and non-genomic signaling interactions

Like ERα, PR exerts non-genomic actions through interactions with intracellular kinases and adaptor proteins: MAPK/ERK and CDK2 phosphorylate PR at Ser294, Ser400, etc, enabling complex formation with SHC, SRC, and PI3K, which triggers cytoplasmic signaling cascades.^[89]^

Key non-genomic pathways include:

*PI3K/AKT pathway*: Progesterone-PR activation of AKT enhances cellular survival, promotes glycolysis, and inhibits apoptotic mechanisms via BCL-2 family modulation. AKT phosphorylation of PR also promotes its nuclear export and reimport, providing feedback regulation.^[89]^

*RANK/RANKL pathway*: PR signaling upregulates RANKL (TNFSF11) expression, particularly in luminal progenitor cells, which facilitates mammary epithelial proliferation and promotes stem cell expansion and basal-like tumor development, particularly in BRCA1 mutation carriers.^[90,91]^ RANKL signaling via its receptor RANK activates NF-κB and AKT pathways, contributing to tumorigenesis. Denosumab, a monoclonal antibody against RANKL, is being studied for cancer prevention and metastasis suppression in high-risk populations.

**Figure 1.**
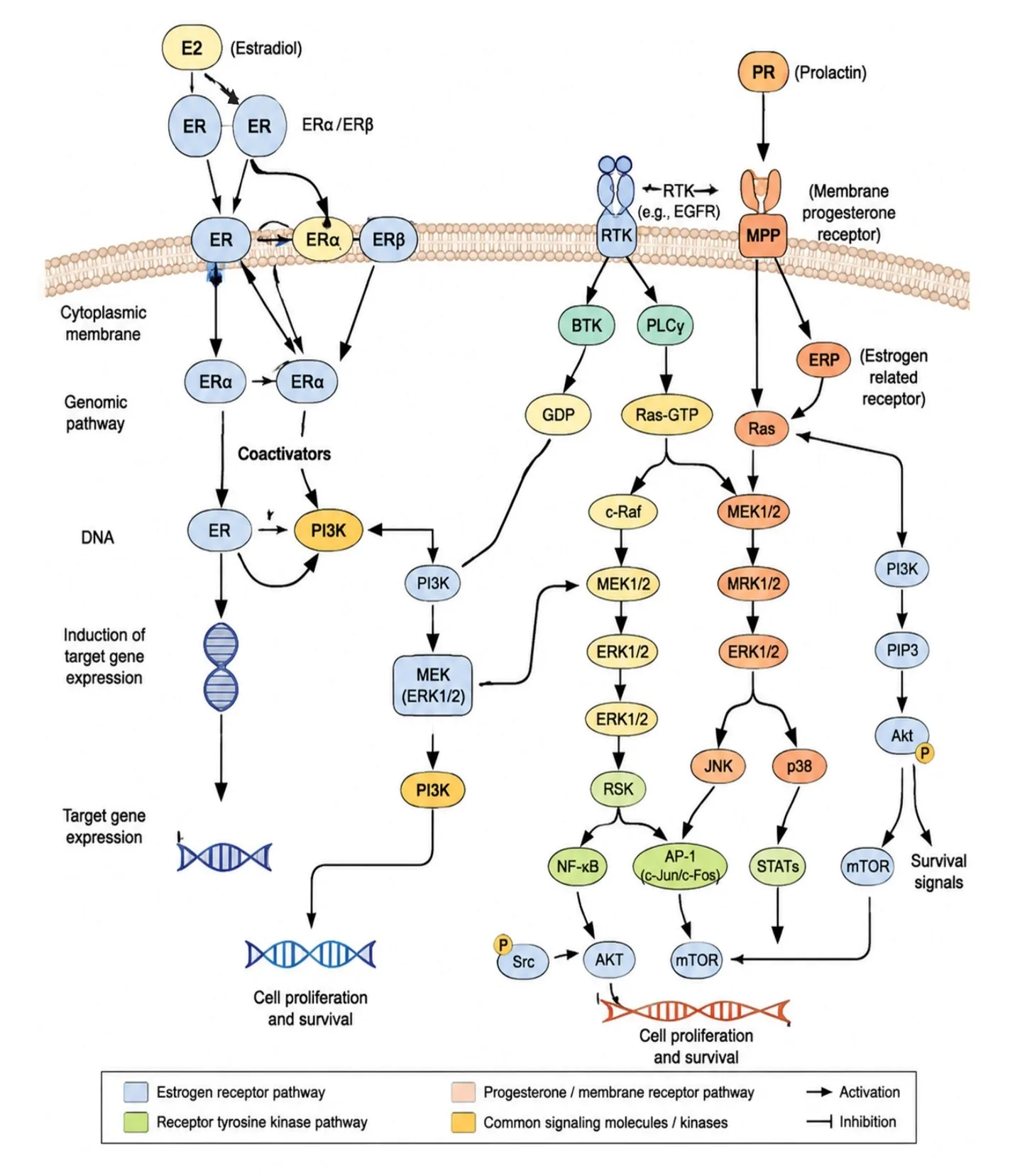
Encapsulates the complex molecular interplay between sex hormone signaling and oncogenic pathways that collectively drive breast cancer initiation, progression, and therapeutic resistance. Rather than depicting estrogen receptor (ER) and progesterone receptor (PR) signaling as isolated linear pathways, the figure illustrates a dynamic signaling network in which genomic and non-genomic hormone actions intersect with growth factor cascades, kinase pathways, and cancer stem cell regulatory circuits.^[43,87-89]^ ER = estrogen receptor, PR = progesterone receptor. Figures 1 & 2 backgrounds were created with the help of an AI tool and recreated/modified using the BioRender app (BioRender. Basajja, M. (2026) https://BioRender.com/y3ldwbj).

The dual mechanisms, genomic (nuclear receptor–mediated transcriptional regulation) and non-genomic (rapid extranuclear signaling), through which estrogen (E2) and progesterone modulate breast cancer cell behavior represent a highly coordinated and dynamic regulatory system. Figure 1 illustrates this integrated signaling network, highlighting how hormone receptor activation interfaces with oncogenic cascades to influence proliferation, survival, invasion, stemness, and therapeutic response. Rather than functioning as isolated linear pathways, genomic and non-genomic mechanisms operate in parallel and reinforce one another, forming a signaling network that underpins hormone-responsive breast cancer biology.

## 9. PI3K/AKT pathway dysregulation in breast cancer

### 9.1. PIK3CA mutations

The PIK3CA gene encodes the p110α catalytic subunit of PI3K. Activating mutations in PIK3CA are among the most common genetic alterations in breast cancer, present in approximately 30% to 40% of cases, particularly in hormone receptor-positive (HR+), HER2-negative subtypes. These mutations lead to constitutive activation of the PI3K/AKT pathway, promoting cell proliferation and survival.^[92]^

### 9.2. PTEN loss

PTEN is a tumor suppressor that negatively regulates the PI3K/AKT pathway by dephosphorylating phosphatidylinositol (3,4,5)-trisphosphate back to PIP2. Loss or inactivation of PTEN – through mutations, deletions, or epigenetic silencing – results in unchecked PI3K/AKT signaling. Such alterations are associated with aggressive tumor behavior and poor prognosis in breast cancer.^[93]^

### 9.3. Non-genomic estrogen activation

Beyond genomic signaling, estrogen can activate the PI3K/AKT pathway through non-genomic mechanisms. ERs, particularly ERα, can associate with the cell membrane and interact with PI3K directly or via adaptor proteins like Src. This rapid activation leads to downstream signaling that promotes cell proliferation and survival, contributing to endocrine resistance.^[94,95]^

#### 9.3.1. Therapeutic implications

Given the central role of the PI3K/AKT pathway in breast cancer pathogenesis, targeting this pathway has become a therapeutic strategy: PI3K Inhibitors: Alpelisib, a PI3Kα-specific inhibitor, has been approved for use in combination with fulvestrant for HR+/HER2− advanced breast cancer with PIK3CA mutations. Clinical trials have demonstrated improved progression-free survival with this combination.^[96,97]^

Combination Therapies: Combining PI3K inhibitors with endocrine therapies may overcome resistance mechanisms. For instance, the addition of inavolisib to palbociclib and fulvestrant has shown promising results in extending overall survival and delaying disease progression in patients with PIK3CA-mutated HR+/HER2− metastatic breast cancer.

**Figure 2.**
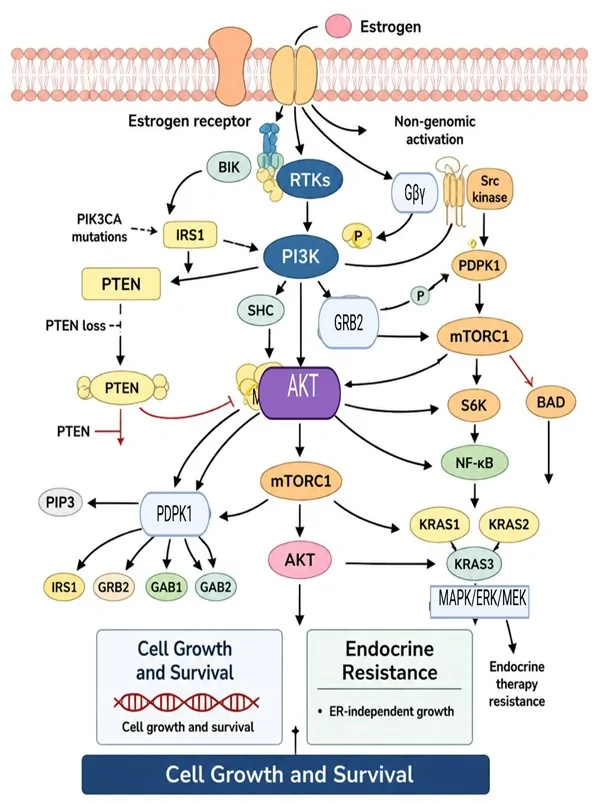
The PI3K/AKT/mTOR signaling pathway is one of the most commonly dysregulated oncogenic pathways in hormone receptor-positive (HR+) breast cancer. Its abnormal activation promotes tumor proliferation, survival, metabolic adaptation, and resistance to endocrine therapy. In many ER-positive tumors, activation results from mutations in the PIK3CA gene, which encodes the p110α catalytic subunit of PI3K. These mutations occur in about 30% to 40% of ER+ breast cancers and drive persistent pathway signaling independent of growth factor control. Activated PI3K generates PIP3, which recruits and activates AKT. AKT then phosphorylates downstream targets including mTOR, FOXO transcription factors, and pro-apoptotic proteins, enhancing protein synthesis and inhibiting apoptosis. AKT can also activate ERα without estrogen, sustaining estrogen-receptor signaling during endocrine therapy and contributing to treatment resistance. Targeting this pathway has become clinically important; the PI3Kα inhibitor alpelisib, combined with fulvestrant, improves outcomes in patients with advanced HR+/HER2− breast cancer harboring PIK3CA mutations, supporting biomarker-guided therapy.^[96,97^^]^ AKT = Protein Kinase B, BAD = BCL2-associated agonist of cell death, BIK = BCL2-interacting killer, ER- Estrogen receptor, ERK = Extracellular signal-regulated kinase, GAB1 = GRB2-associated binding protein 1, GAB2 = GRB2-associated binding protein 1, Gβγ = G-protein beta-gamma subunit, GRB2 = Growth factor receptor-bound protein 2, IRS1 = Insulin receptor substrate 1, KRAS = Kirsten rat sarcoma viral oncogene homolog, MAPK = Mitogen-activated protein kinase, MEK = Mitogen-activated protein kinase kinase, mTORC1 = Mechanistic target of rapamycin complex 1, NF = κB, Nuclear factor kappa-light-chain-enhancer of activated B cells, PDPK1/PDK1 = 3-phosphoinositide-dependent protein kinase 1, PI3K = Phosphoinositide 3-kinase, PIK3CA = Phosphatidylinositol-4,5-bisphosphate 3-kinase catalytic subunit alpha, PIP3 = Phosphatidylinositol-3,4,5-trisphosphate, PTEN = Phosphatase and tensin homolog, RTKs = Receptor tyrosine kinases, SHC = Src homology 2 domain-containing transforming protein, S6K = Ribosomal protein S6 kinase, Src-kInase = Sarcoma-family tyrosine kinase.

Figure 2 illustrates how estrogen signaling, *PIK3CA* mutations, and PTEN loss converge to hyperactivate the PI3K/AKT pathway in breast cancer cells, creating a central oncogenic axis that drives cell growth, survival, metabolic adaptation, and endocrine resistance. Rather than functioning as isolated molecular abnormalities, these alterations act cooperatively to produce sustained and amplified PI3K/AKT signaling. The figure conceptualizes this convergence as a multi-input activation model in which hormonal, genetic, and tumor suppressor pathway disruptions collectively reinforce pathway hyperactivation.

Functions and clinical relevance of dysregulation of the PI3K/AKT Pathway in Breast Cancer are summarized in Table 1.

**Table 1:** Functions and clinical relevance of dysregulation of the PI3K/AKT pathway in breast cancer.^[96,97]^

Component	Function	Clinical relevance
Estrogen	Activates non-genomic PI3K/AKT pathway	Contributes to endocrine resistance
PIK3CA	Mutations cause constitutive PI3K activation	Found in ~40% of HR+ breast cancers
PTEN	Tumor suppressor that blocks PI3K/AKT	Loss leads to pathway hyperactivation
AKT	Promotes survival and antiapoptotic signaling	Central driver of tumor aggressiveness
Outcome	Proliferation, survival, endocrine resistance	Key therapeutic target in modern oncology

HR+ = hormone receptor-positive, PI3K/AKT = phosphoinositide 3-kinase/protein kinase B, PTEN = phosphatase and tensin homolog.

## 10. HER2 overexpression and MAPK/ERK activation

HER2 is overexpressed in approximately 15% to 30% of breast cancers, leading to aggressive tumor behavior and poor prognosis. HER2 overexpression results in the formation of HER2 homodimers and heterodimers, particularly with HER3, which robustly activate downstream signaling cascades, including the MAPK/ERK pathway. This activation promotes cell proliferation and survival, contributing to tumorigenesis.

### 10.1. Estrogen interaction with the MAPK/ERK pathway

Estrogen exerts both genomic and non-genomic effects in breast cancer cells. Non-genomic actions involve the activation of membrane-associated estrogen receptors, leading to rapid signaling events. Specifically, estrogen can activate the MAPK/ERK pathway through interactions with receptor tyrosine kinases and downstream effectors. This activation enhances cell proliferation and may contribute to resistance against endocrine therapies.^[98,99]^

### 10.2. Persistent MAPK/ERK activation and therapy resistance

Continuous activation of the MAPK/ERK pathway is associated with increased tumor growth and resistance to therapies targeting ER signaling. Mechanisms contributing to this persistent activation include – Loss of negative regulators: downregulation or loss of dual-specificity phosphatases (DUSPs), such as DUSP7, leads to sustained ERK activation. Long non-coding RNAs (lncRNAs): LncRNAs like linc-RoR have been shown to promote estrogen-independent growth and tamoxifen resistance by activating the MAPK/ERK pathway. Linc-RoR facilitates the degradation of DUSP7, thereby sustaining ERK activation and promoting ligand-independent activation of ER signaling.^[100]^

### 10.3. Therapeutic implications

Targeting the MAPK/ERK pathway presents a promising strategy in overcoming resistance in breast cancer therapy. MEK inhibitors, such as binimetinib, have been developed to inhibit this pathway. However, monotherapy with MEK inhibitors has shown limited efficacy due to compensatory activation of parallel pathways. Combination therapies targeting both MAPK/ERK and PI3K/AKT pathways are being explored to enhance treatment efficacy and overcome resistance mechanisms.^[101]^

**Figure 3.**
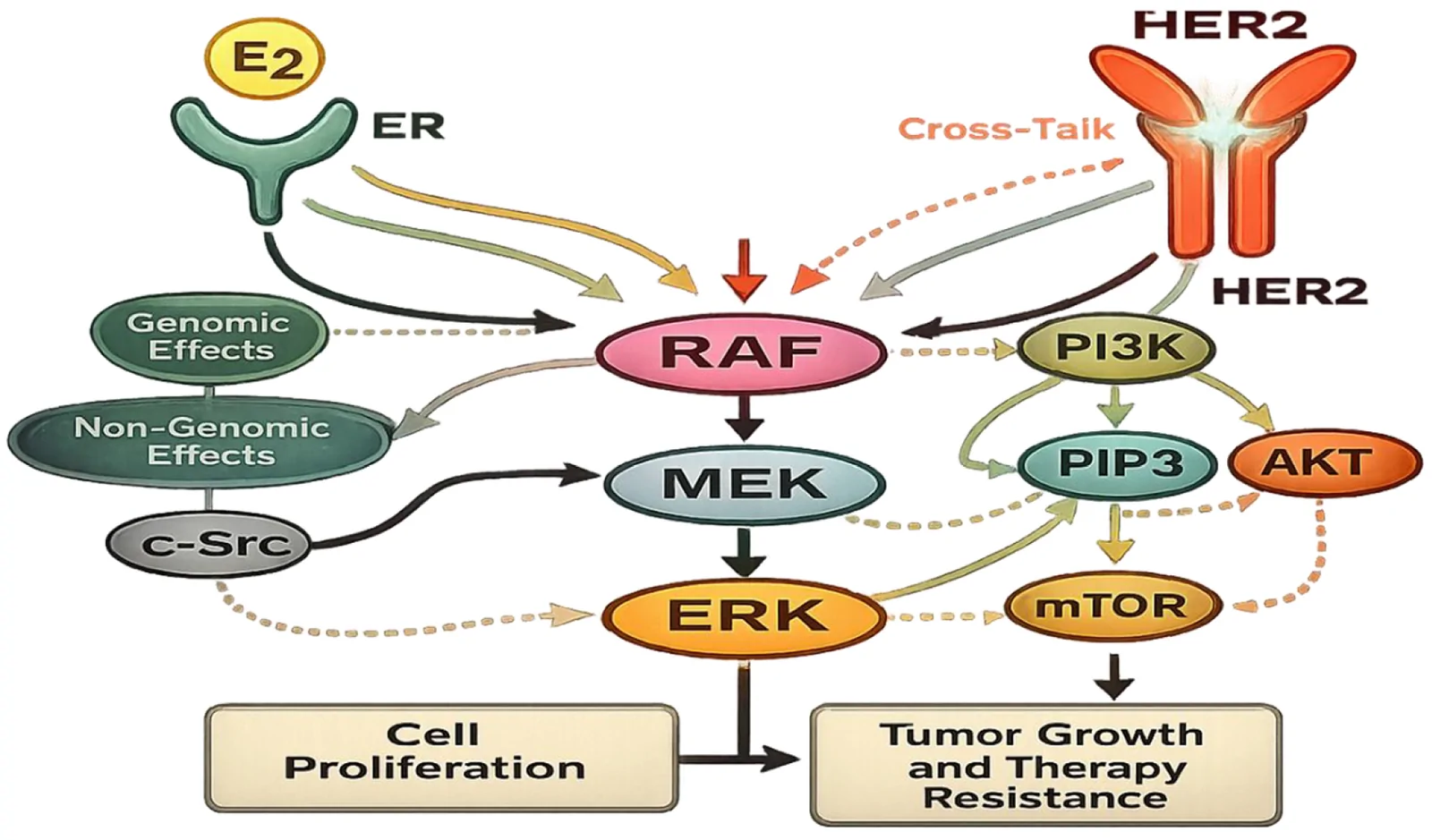
Illustrates how HER2 overexpression and non-genomic estrogen signaling converge on the MAPK/ERK pathway, creating a potent mitogenic axis that enhances gene transcription, cellular proliferation, and therapeutic resistance in breast cancer cells. Rather than functioning independently, HER2-driven growth factor signaling and membrane-initiated estrogen signaling form a cooperative signaling network that amplifies downstream kinase activation. The convergence of these pathways at the level of MAPK/ERK represents a critical node of oncogenic integration in both HER2-enriched and certain hormone receptor–positive breast cancers.^[101]^ ER = estrogen receptor, ERK = extracellular signal-regulated kinase, HER2 = human epidermal growth factor receptor 2, MAPK = mitogen-activated protein kinase, MEK = mitogen-activated protein kinase kinase, mTOR = mechanistic target of rapamycin, PI3K/AKT = phosphoinositide 3-kinase/protein kinase B, PIP3 = phosphatidylinositol (3,4,5)-trisphosphate.

Figure 3 illustrates how HER2 overexpression and non-genomic estrogen signaling converge on the MAPK/ERK pathway, forming a central proliferative signaling axis that enhances transcriptional activation and drives breast cancer cell growth. This convergence represents a critical example of oncogenic pathway integration, where growth factor receptor signaling and steroid hormone signaling cooperate rather than function independently. The MAPK/ERK cascade serves as a molecular integration hub, amplifying mitogenic output and reinforcing tumor progression.

Clinical implications of HER2 overexpression and non-genomic estrogen signaling converge on the MAPK/ERK pathway, ultimately enhancing gene expression and proliferation in breast cancer cells, as summarized in Table 2.

**Table 2:** Clinical implications of MAPK/ERK pathway dysregulation in breast cancer.^[101]^

Dysregulated component	Effect on cancer	Clinical consequence
HER2 overexpression	ERK activation	Resistance to trastuzumab or lapatinib
ER–MAPK crosstalk	Redundant survival signaling	Endocrine resistance
Sustained ERK signaling	Uncontrolled gene expression	Tumor progression, metastasis

ER = estrogen receptor, ERK = extracellular signal-regulated kinase, HER2 = human epidermal growth factor receptor 2, MAPK = mitogen-activated protein kinase.

## 11. Crosstalk between hormonal and intracellular signaling pathways in breast cancer

The progression of breast cancer and the emergence of resistance to therapy are not governed by a single signaling pathway but rather by a complex network of interactions – or “crosstalk” – between hormonal signaling and intracellular pathways. Estrogen and progesterone, acting through their respective nuclear receptors, modulate key signaling cascades such as PI3K/AKT, MAPK/ERK, and Notch, which together contribute to tumor proliferation, survival, CSC maintenance, and therapy resistance.

Estrogen exerts many of its proliferative effects in hormone receptor-positive breast cancer by activating PI3K/AKT signaling. Estrogen binds to ERα, which can initiate non-genomic signaling via membrane-localized ERs. This activates PI3K either directly or through intermediate proteins like Src and insulin-like IGF-1R. The subsequent phosphorylation cascade involving AKT enhances cell survival, cell cycle progression, and resistance to apoptosis.^[102]^

In addition to PI3K/AKT, estrogen also stimulates the MAPK/ERK pathway through rapid, non- genomic mechanisms. Upon binding to membrane-bound ERs or interacting with GPER, estrogen activates Ras/Raf, which subsequently activates MAPK/ERK signaling. This leads to transcriptional activation of genes involved in proliferation (e.g., c-Fos, Elk-1) and cell motility.^[103]^

Understanding the interconnectedness of hormonal and intracellular signaling is vital for effective breast cancer treatment strategies. Monotherapies targeting ER or PR alone are often insufficient due to compensatory activation of survival pathways like PI3K/AKT. As such, dual or multi- targeted therapies (e.g., ER blockers + PI3K inhibitors) show promise in overcoming resistance.

Examples include Alpelisib (PI3Kα inhibitor) in ER+ breast cancer with PIK3CA mutations, currently under clinical evaluation in combination with endocrine therapies.^[98]^

**Figure 4.**
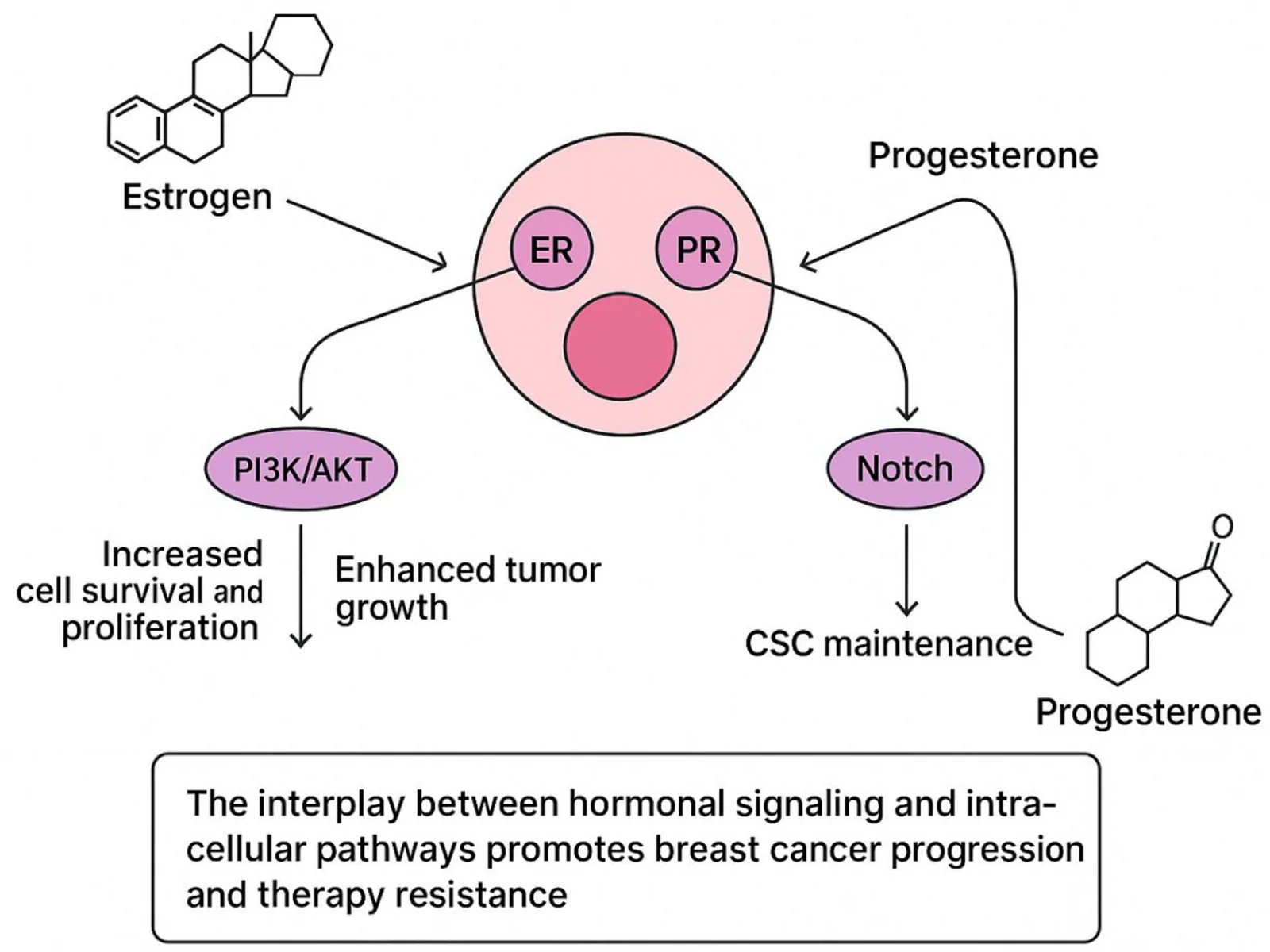
This figure illustrates the visual synthesis of the interconnected signaling networks between hormonal receptors (estrogen and progesterone) and key intracellular pathways (PI3K/AKT, MAPK/ERK, and Notch) that contribute to breast cancer progression, therapy resistance, and cancer stem cell (CSC) maintenance.^[102,103]^ CSC = cancer stem cell, ER = estrogen receptor, ERK = extracellular signal-regulated kinase, MAPK = mitogen-activated protein kinase, PI3K/AKT = phosphoinositide 3-kinase/protein kinase B, PR = progesterone receptors.

A visual synthesis of the interconnected signaling networks between hormonal receptors (estrogen and progesterone) and key intracellular pathways (PI3K/AKT and MAPK/ERK) that contribute to breast cancer progression, therapy resistance, and cancer stem cell (CSC) maintenance is illustrated in Figure 4.

## 12. Interconnected signaling networks

The interaction of hormone receptors with pertinent signaling, such as PI3K-Akt and MAPK, reveals complex relationship exhibiting the molecular profile of breast cancer. Such relationships have to be explained to reveal the general effects of sex hormones in detail. Many of the cellular signaling pathways are intertwined and thus communicate to form complex signaling pathways, meaning the activator of 1 signaling pathway influences other pathways. For instance, a cell proliferation pathway known as the MAPK pathway may connect with another pathway, the PI3K-AKT, that influences both growth and survival.^[104,105]^ Both ER and GR integrate with other signal transduction pathways to modulate gene expression of target tissues. ER can communicate with growth factor signaling systems and affect cell division, and GR can modulate inflammatory signaling systems.^[106]^

The PI3K-AKT-mTOR pathway is activated by a large number of receptor populations such as the growth factor receptor and the hormone receptor, as well as the receptors that sense nutrient availability. One example of such a kinase is AKT, which constitutes a signaling crossroad receiving signals from multiple downstream pathways.^[107,108]^ MAPK/ NF-κB pathways are very interconnected and they regulate several results of inflammation, stress and immune signals in the cell. MAPKs can phosphorylate the core participants, which also connect NF-κB as part of a complex signaling web.^[109]^

## 13. Cross-talk between hormone receptors and signaling pathways

ER and PR are interconnected with other signaling pathways, and these include those with growth factor signaling, and the PI3K-Akt-mTOR pathway, making regulation more complex in breast cancer cell behaviors.^[36]^ ER signaling interacts with other signaling pathways like the receptor, e.g., epidermal growth factor receptor (EGFR) and insulin-like growth factor receptor (IGFR) concerning cell growth and survival. ER can be activated by ligands, such as estrogens, participating in the cross-talk with growth factor signaling; it can also activate downstream kinases, which are MAPK and AKT.^[110]^

Testosterone-AR/PI3K-AKT axis is relevant to the disease progression of PCa. Recent data have shown that androgens can also activate AKT and hence promote cell survival and proliferation of prostate cancer cells.^[111]^ Lef:Thr can either stimulate or inhibit the activity of β-catenin in determining cell characteristics and tissue repair.^[112]^ The NF-κB pathway is an important regulator of immune and inflammation: GR can suppress NF-κB activation in some settings.^[113]^ ER functions as cross-talked pathways stimulating mammary gland formation and modulating breast cancer processes.^[114]^

## 14. Therapeutic approaches for breast cancer

Tamoxifen coming under selective estrogen receptor modulators (SERMs), works by binding only to the estrogen receptors (ER) in certain tissues. SERMs are used as estrogen receptor antagonists in breast tissue where they counteract estrogen’s mitogenic effects and arrest tumor formation. In bone tissue SERMs can be estrogen agonist and can help to maintain close density.^[115]^ Selective estrogen receptor modulators (SERMs), including tamoxifen and raloxifene, act by binding to the estrogen receptor and then blocking the receptor with the estrogen that is present in the blood. Because AIs act on the ovaries to decrease estrogen production, these drugs decrease the levels of estrogen in the circulation; therefore, affecting hormone-dependent tumor development. AIs are most helpful in postmenopausal women where the majority of estrogens come from aromatase.^[116]^

The new strategies that have been developed for treatment of BC, especially those targeting the androgen receptor and other hormonal pathways, are complex at a molecular level. These approaches are intended to block or regulate AR expression and/or activity and/or modulate the expression and activity of other hormones or hormone receptors and pathways including the ER, PR, and growth factor signaling pathways. It is, therefore, important that the molecular process governing these strategies is well understood so that discerning therapeutic approaches can be targeted in breast cancer.^[117]^

One of the key molecular mechanisms is the inhibition of AR signaling. ARs are known to be present in some types of breast cancer and do play a role in cancer development and progression. AR activation comprises the interaction of non-aromatizable androgens, including testosterone and dihydrotestosterone (DHT), with the AR, resulting in dimerization and activation of respective intracellular signal cascades associated with cell growth and survival promotion. The following strategies work on eliminating or reducing AR signaling: The first method involves using selective AR modulators (SARMs), which block activation of AR; the second method is through androgen synthesis suppression by using antiandrogen drugs such as abiraterone. These approaches have demonstrated potential in preclinical and very early phase clinical trials, thus reducing tumor cell proliferation and mortality rates.^[117,118]^

Besides AR signaling inhibition, new modalities are mostly based on targeting several hormonal signaling pathways at a time. For instance, combination therapy that involves the targeting of ER and AR signaling appeared to be very effective, where both ER and AR are positive in breast cancer patients’ treatments targeting ER and AR show better effectiveness in suppressing tumor as well as in improving patient prognosis.^[119]^

Additionally, the goal of targeting hormone-related pathways also incorporates approaches to avoid resistance to treatment mechanisms. For example, in metastatic ER-positive breast cancer patients that fail endocrine treatment, different factors can influence treatment failure: changes in ER signaling, cross-talk with growth factor receptors, and activation of additional signaling. These resistance mechanisms can be overcome by designing drugs that will block multiple signaling pathways as a single agent or by gaining further understanding about molecules that uniquely interact with these altered signaling pathways.^[120]^

## 15. Conclusion

This outstanding work has offered a coherent description regarding complex molecular processes with regard to the contribution of sex hormones in breast cancer development. Hence, by teasing out the roles of estrogen, progesterone, their receptors, and androgens in breast cancer management, this review has explained key signaling pathways, gene expressions, and cell processes in breast cancers. Any such understanding becomes very important for defining new diagnostic markers, new targets for therapies, and efficient treatments. Furthermore, it emphasizes the need for treatment modalities that incorporate heuristic models regarding hormone receptor status and pathobiological changes occurring in breast cancer.

## 16. Article summary

Unraveling the intricate molecular mechanisms of sex hormones’ roles in breast cancer pathogenesis, “the authors explore the complex ways in which sex hormones contribute to breast cancer development. The choice of the areas is justified by the current state of molecular and cellular biology where there is much interest in the mechanisms of actions of sex hormones and their receptors, the crosstalk with signaling pathways, the impact of noncoding RNA, and the regulation of sex hormones metabolism. Some possible therapeutic approaches were considered concerning the interference with sex hormone signaling in the breast cancer context. From the current study, a general knowledge of the complex molecular processes that govern the development of breast cancer through steroid receptor signaling is gained.

## 17. Strengths and limitation

The article provides a comprehensive overview of how sex hormones influence breast cancer pathogenesis, covering hormone-receptor interactions, signaling pathways, and noncoding RNAs. It highlights recent advances and detailed molecular interactions, aiding in therapeutic strategy identification. However, its complex nature may challenge readers without molecular biology expertise. The review emphasizes specific studies or models, potentially limiting generalizability across all breast cancer subtypes and populations. There may be biases stemming from the authors’ research perspectives, possibly excluding alternative viewpoints. Readers should critically evaluate the findings alongside other sources for balanced insights into sex hormone-mediated breast cancer mechanisms and implications.

## 18. Recommendation

Future investigations should focus on integrating advanced technologies, such as single-cell sequencing and functional genomics, to unravel finer details in order to advance targeted therapeutic strategies and personalized treatment approaches for breast cancer patients.

## Author contributions

**Conceptualization:** Matthew Chibunna Igwe.

**Supervision:** Matthew Chibunna Igwe.

**Writing – original draft:** Matthew Chibunna Igwe.

**Writing – review & editing:** Matthew Chibunna Igwe, Ogbonna Alphonsus Ogbuabor, Simeon Ikechukwu Egba.
