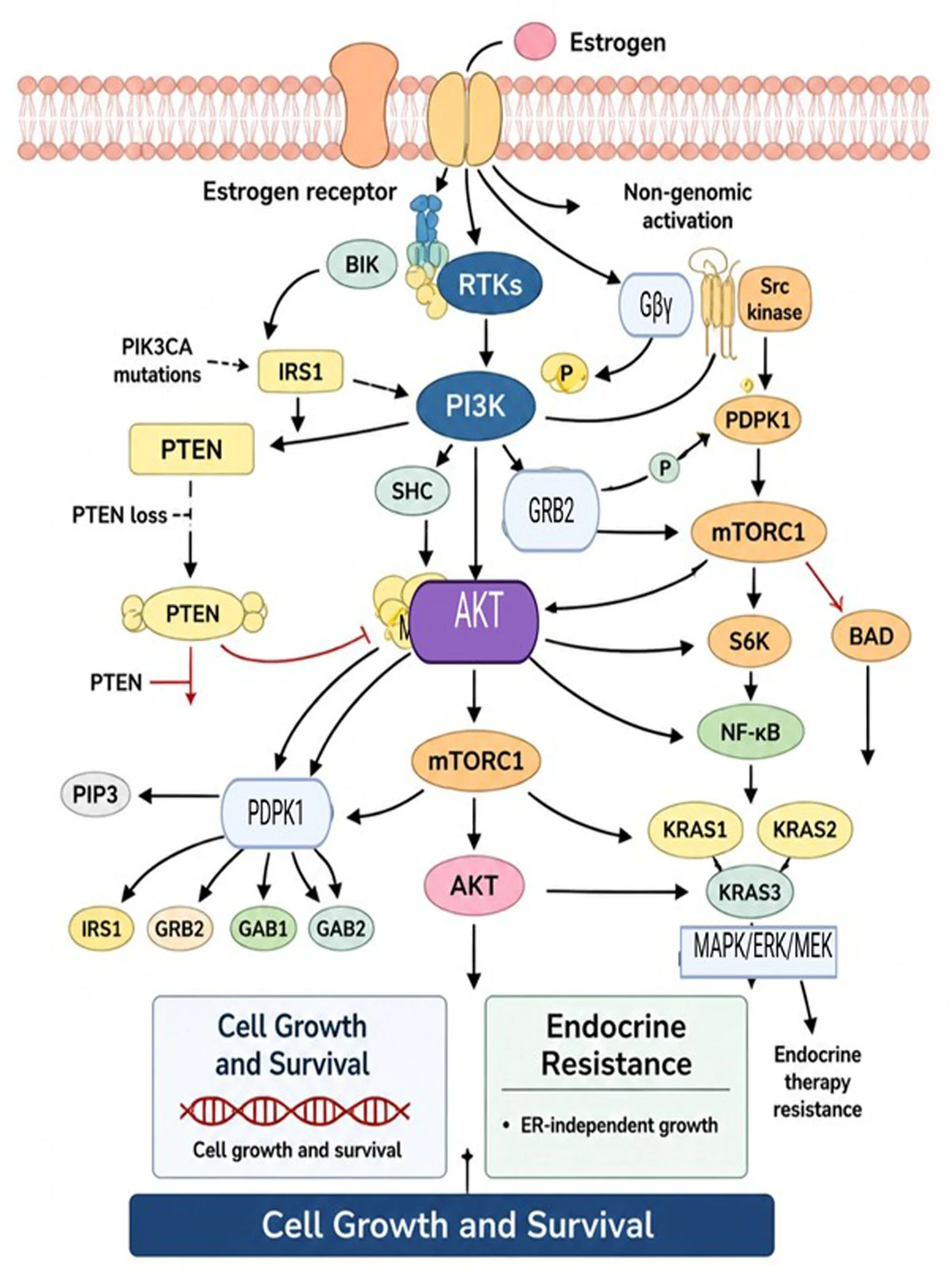# Unraveling the intricate molecular mechanisms of sex hormones’ roles in breast cancer pathogenesis. A review: Erratum

**DOI:** 10.1097/MD.0000000000050689

**Published:** 2026-09-18

**Authors:** Matthew Chibunna Igwe, Ogbonna Alphonsus Ogbuabor, Simeon Ikechukwu Egba

In the article “Unraveling the intricate molecular mechanisms of sex hormones’ roles in breast cancer pathogenesis: A review”,^[1]^ which appears in Volume 105, Issue 26 of *Medicine*, incorrect figures 1 and 2 were inadvertently published. The complete versions of figures 1 and 2 have now been updated in the published article.

**Figure 1:**
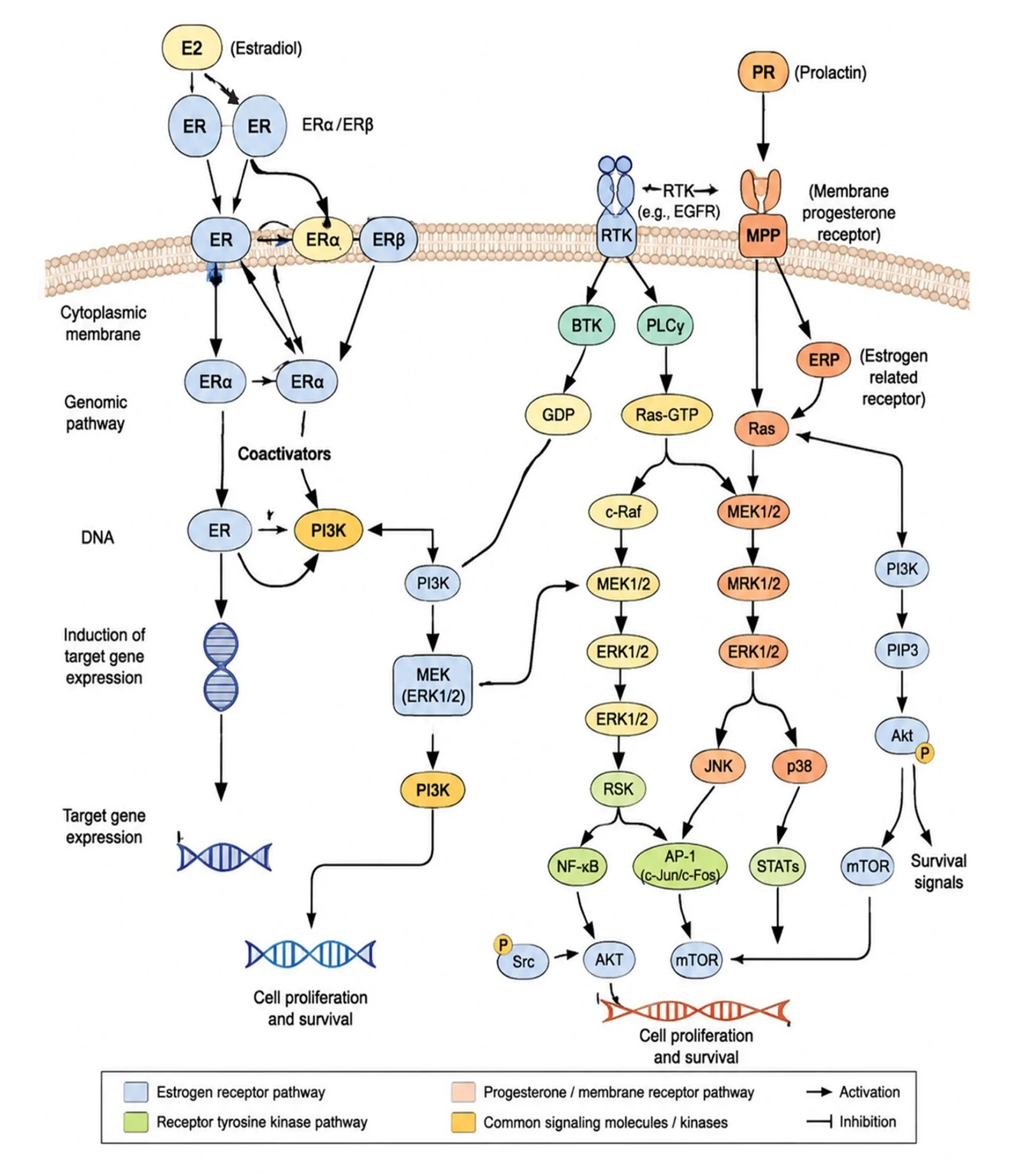


**Figure 2:**